# Supplementary material for: Learning curve and surgical outcome of robotic assisted colorectal surgery with ERAS program
Source: Sci Rep. 2022 Nov 29;12:20566. doi: 10.1038/s41598-022-24665-w (PMC9709162; doi:10.1038/s41598-022-24665-w)
Supplement: Supplementary file 1 — Supplementary Information 1. [file 41598_2022_24665_MOESM1_ESM.zip › LSC Raw data-Ñ[▒K/130/H_ñw╝╨░O▒Kñσ.pdf]

正式摘要(電子簽章：000000221109465)

W52 / W52 ☐英文標題(注意:無英文姓名-請補病人基本檔!) ☐【讀取】憑證

臺中榮民總醫院

出院病歷摘要

轉入號： 轉出號：

[redacted]

(8)流水編號	
(9)入院日期	110/07/14 (2021) 直腸外科 W52 - 161
(10)轉科日期	
(11)出院日期	110/07/21 (2021) 住院天數計 7 日

診斷

- 入院 :1. Upper rectal adenocarcinoma, 10cm FAV, s/p colonscopic biopsy at Kuang Tien General hospital
  2. PPU, s/p operation at 李綜合hospital for 10+ years
  3. CVA, infarction, in 2020, s/p medication for 6+ months
  4. Alzheimer's disease, diagnosed in 2020, under medication
  5. HTN, diagnosed in 2020, under medication
  6. BPH, diagnosed in 2020, under medication
  7. Hemorrhoid, s/p operation at Kuang Tien General hospital in 2021
  8. Bilateral hearing impairment
- 出院 :1. Upper rectal adenocarcinoma, 10cm FAV, s/p colonscopic biopsy at Kuang Tien General hospital, s/p laparoscopic-converted-to-conventional AR on 2021/7/15
  2. Multiform VPCs with non-sustained VT(+), LVEF 46%, under Herbesser 3. PPU, s/p operation at 李綜合hospital for 10+ years
  4. CVA, infarction, in 2020, s/p medication for 6+ months
  5. Alzheimer's disease, diagnosed in 2020, under medication
  6. HTN, diagnosed in 2020, under medication
  7. BPH, diagnosed in 2020, under medication
  8. Hemorrhoid, s/p operation at Kuang Tien General hospital in 2021
  9. Bilateral hearing impairment

主訴

- loose stool with blood-tinged intermittently for several months

病史

- This 72-year-old man has past history of:
  1. Upper rectal adenocarcinoma, 10cm FAV, s/p colonscopic biopsy at Kuang Tien General hospital
  2. PPU, s/p operation at 李綜合hospital for 10+ years
  3. CVA, infarction, in 2020, s/p medication for 6+ months
  4. Alzheimer's disease, diagnosed in 2020, under medication
  5. HTN, diagnosed in 2020, under medication
  6. BPH, diagnosed in 2020, under medication
  7. Hemorrhoid, s/p operation at Kuang Tien General hospital in 2021
  8. Bilateral hearing impairment

  According to his wife, he presented with loose stool with blood-tinged intermittently for several months, which was approximately the time after discharge from other hospital (admission for stroke).
  He visited Kuang Tien General hospital at first, wherer hemorrhoid was diagnosed, s/p operation. However, the bloody stool did not improved. Thus, he received UGI+colonscopy, which biopsy at 10cm FAV reported adenocarcinoma.
  He then came to our hospital. Tumor markers was checked, with CEA 18.87 ng/ml and undetectable CA-199.
  Abdominal CT on 2021/06/23 showed S-colon cancer (5.1cm) with pericolic lymphadenopathy (2.4cm-length LN).
  PET on 2021/07/01 revealed tumor at presacral region (Grade 4).
  Under the impression of upper rectal adenocarcinoma with pericolic lymphadenopathy, 10cm FAV, biopsy proved, the patient was admitted for laparoscopic AR scheduled on 2021/07/15.

體檢發現

-
  - General appearance :
    - easy looking , conscious clear , without cardiopulmonary distress
    -

-
    - Vital signs自動帶入date 體溫(BT) 脈搏(P) 呼吸(R) 血壓(BP) 身高(BH) 體重(BW) BMI
      20210714 36℃ 45次/分 19次/分 177/103mmHg 153cm 68.6kg 29.3
    - Vital signs: B.P. 177/103 mmHg , P.R. 45 /min , R.R. 19 /min , B.T. 36 ℃
- HEENT :
    - Eyes: no anemic conjunctiva, no icteric sclera, normal EOM
    - ENT: no otorrhea, no rhinorrhea, no erythematous mucosa, no injected tonsil, no deformity
- Neck: supple, freely movable, trachea midline,no deformity, no engorged jugular vein , no palpable thyroid or lymph node enlargement
- Chest:
    - Inspection: Grossly normal, symmetric and fully expansion, no accessory muscle used, no operation scar, no spider angiomata, no gynecomastia
    - Palpation: no palpable mass or nodule, no subcutaneous emphysema, no tenderness
    - Ausculation: clear breathing sound
- Heart:
    - Palpation: no thrill, no heave
    - Percussion: normal heart size
    - Ausculation: regular heart beat, normal S1, S2, no gallop, no murmur
- Extremities: No deformity, full ranged of motion without limitation, no clubbing finger or nail cyanosis, no pitting edema, no ulcer or pigmentation, no obvious skin lesion, no varicose vein, no bone, joint or muscle tenderness

CRS CONDITION

- Karnofsky performance status: (100) Normal, no complaints, no evidence of disease
- PE of Abdomen:
- CEA: 18.87 ng/ml
- Imaging Studies:
- Colonoscope report: UGI+colonscopy, which biopsy at 10cm FAV reported adenocarcinoma at Kuang Tien General hospital
- Sono , CT of abdomen and chest Films:Abdominal CT on 2021/06/23 showed S-colon cancer (5.1cm) with pericolic lymphadenopathy (2.4cm-length LN).
  PET on 2021/07/01 revealed tumor at presacral region (Grade 4).
- Figures:
    1. colon & rectum

手術日期及方法

- 手術日期及方法20210715 1. Laparoscopic low anterior resection convert to open due to severe adhision due to PPU s/p OP >10yrs
  , end-to-end anastomosis, TA 45/4.8mm, CEEA- 33mm. Bilateral doughnut: intact, air tight test: done, no air leak.
  2. High ligation (+) above the left colic artery.
  3. Take down of splenic flexure: (-).
  4. A 12mm penrose drain placed in the pelvis.
  5. Surgiwrap +

住院治療經過

- After operation with AR on 2021/07/15, the patient was under post-operative care and pain control under PCA use. Intra-operative arrhythmia with frequent VPCs was noted during initiation of high-flow CO2 insufflation for laparoscopy. AR was converted from laparoscopy to conventional surgery due to anesthesia concern and severe adhesion. Self-urination was smoothly after removal of Foley's catheter on 7/17. Penrose drain was removed on 7/19. The patient tolerated clear liquid diet and then Bland No.II diet since 7/17, No.III since 7/19, without N/V, abdominal pain, but mild abdominal fullness. The wound was clean and dry, without infectious signs; stitches were removed on 7/21. For VPCs, CV was consulted. Very high burden multiform VPCs (>10-20%) and non-sustained VT(+) was noted by Holter's ECG. 2D-echo reported reduced LVEF as 46% and mild generalized hypokinesia of LV. CAG/PCI, cardiac MRI and EPS/ABL were suggested, but the family refused. The risks of syncope and sudden cardiac death were informed. Herbesser was added; CV OPD follow up was suggested. Under relative stable condition, the patient was discharge with OPD follow up.

合併症

一般檢查

- 累積報告 :

DATE	TIME	ALB	TP	BIL.T	BIL.D	ALKP	AST	ALT	LDH	AMY	LIPASE	NH3	Ferritin	UA

1100714	1847	3.9		0.5			18	11						
1100715	1820	3.4		0.87			19	13						
1100719	0851	2.9		0.59			11	8						

DATE	TIME	WBC	RBC	HGB	HCT	MCV	PLT	BLST	NEUT	BAND	SEG	LYM	MON	EOS	BAS
1100714	1846	7230	4.73	13.5	41.8	88.4	230		52.8			35.8	7.6	3.2	0.6
1100715	1819	11670		11.2	35.6	89.0	190		90.4			5.1	4.4	0.0	0.1
1100719	1000	9210		11.2	34.6	88.7	166		71.4			17.9	7.7	2.8	0.2

DATE	TIME	NA	K	CL	BUN	CREAT	eGFR	CA	Mg	P	CRP	ESR	PCT	D-Dimer	LACTATE
1100714	1847	144	3.9		20	1.01		8.7							
1100715	1819														11.2
1100715	1820	142	3.7		11	0.86	92.91	8.0							
1100719	0851	143	3.3		16	1.04	74.61	8.1	2.0	2.3					
1100719	0905											19			
1100719	0916													4.05	

DATE	TIME	GLUCOSE	HbA1C	CHOL	TG	HDL	LDL	KETONE	CK	CKMB	TRO-T	PT-P	APTT-P	NT-pro BNP
1100715	1819										12.15			
1100715	1820								130	11				
1100719	0901													6868.00
1100719	0916											11.8	35.3	

DATE	TIME	PH	BEB	SCVO2	PO2	HCO3	HGB	PCO2	TCO2
1100715	1816	7.380	-0.3	97.2	96.0	25.0		43.4	26.4

特殊檢查

病理報告

其它

出院時情況

- 3.改門診治療

出院指示

藥名	劑量	劑量單位	用法	頻次	數量	數量單位	現況	天數	單位
(口)Takepron OD tab 30mg	1	TAB	PO	QDAC	7	TAB	TH	7	天
Ultracet tab	1	TAB	PO	Q6H	28	TAB	TH	7	天
Acetylcysteine tab	1	TAB	PO	BID	14	TAB	TH	7	天
Econ tab 40mg	1	TAB	PO	QID	28	TAB	TH	7	天
(口)Rosis tab 40mg	1	TAB	PO	BID	14	TAB	TH	7	天
Herbesser tab 30mg	1	TAB	PO	TIDAC	21	TAB	TH	7	天

出院計劃

- 門診日期
  - date on 2021/07/27
- Herbesser was added; CV OPD follow up was suggested.

主治醫師 2062F 蔣鋒帆 / 住院醫師 2345B 陳映晴

正式摘要(電子簽章：000000221109465)

W52 / W52

doctorRank:B
